# Identification and verification of inflammatory biomarkers for primary Sjögren’s syndrome

**DOI:** 10.1007/s10067-024-06901-y

**Published:** 2024-02-20

**Authors:** Xiaodan Liu, Haojie Wang, Xiao Wang, Xiaodan Jiang, Yinji Jin, Ying Han, Zhihui Zhang

**Affiliations:** 1 Department of Stomatology, Peking University Third Hospital, Haidian District, 49 North Garden Road, Beijing, 100191 China; 2 Department of Ophthalmology, Beijing Key Laboratory of Restoration of Damaged Ocular Nerve, Peking University Third Hospital, Peking University Third Hospital, Beijing, 100191 China; 3 Department of Rheumatology and Immunology, Peking University Third Hospital, Beijing, 100191 China; 4 Department of Oral Medicine, Laboratory for Digital and Material Technology of Stomatology & Beijing Key Laboratory of Digital Stomatology, Peking University School and Hospital of Stomatology & National Clinical Research Center for Oral Diseases & National Engineering, Haidian District, 22 Zhongguancun South Avenue, Beijing, 100081 China

**Keywords:** Biomarkers, Immune infiltration cell, Inflammatory-associated genes, Primary Sjögren’s syndrome

## Abstract

**Introduction:**

Primary Sjögren’s syndrome (pSS) is an autoimmune disease characterized by inflammatory infiltration, and dysfunction of the salivary and lacrimal glands. This research aimed to explore the disease pathogenesis and improve the diagnosis and treatment of pSS by mining inflammation-associated biomarkers.

**Methods:**

Five pSS-related datasets were retrieved from the Gene Expression Omnibus (GEO) database. Inflammation-associated biomarkers were determined by the least absolute shrinkage and selection operator (LASSO) and support vector machines recursive feature elimination (SVM-RFE). Single sample gene set enrichment analysis (ssGSEA) was implemented to profile the infiltration levels of immune cells. Real-time quantitative PCR (RT-qPCR) verified the expression of biomarkers in clinical samples.

**Results:**

Four genes (LY6E, EIF2AK2, IL15, and CXCL10) were screened as inflammation-associated biomarkers in pSS, the predictive performance of which were determined among three pSS-related datasets (AUC > 0.7). Functional enrichment results suggested that the biomarkers were involved in immune and inflammation-related pathways. Immune infiltration analysis revealed that biomarkers were notably connected with type 2 T helper cells, regulatory T cells which were significantly expressed between pSS and control. TESTOSTERONE and CYCLOSPORINE were predicted to take effect by targeting CXCL10 and IL15 in pSS, respectively.

**Conclusion:**

Four inflammation-associated biomarkers (LY6E, EIF2AK2, IL15, and CXCL10) were explored, and the underlying regulatory mechanisms and targeted drugs associated with these biomarkers were preliminarily investigated according to a series of bioinformatics methods based on the online datasets of pSS, which provided a reference for understanding the pathogenesis of pSS.

**Table:** 

Key Points • *Inflammation-associated biomarkers (LY6E, EIF2AK2, IL15, and CXCL10) were firstly identified in Sjögren’s syndrome based on LASSO and SVM-RFE analyses.* • *CXCL10, EIF2AK2 and LY6E were prominently positively correlated with immature B cells, while IL15 were significantly negatively correlated with memory B cells in Sjögren’s syndrome.* • *LY6E, EIF2AK2, IL15, and CXCL10 were significantly more highly expressed in clinical Sjögren’s syndrome samples compared to healthy control samples, which was consistent with the analysis results of the GEO database.* •*LY6E, EIF2AK2, IL15, and CXCL10 might be used as the biomarkers for the treatment and diagnosis of Sjögren’s syndrome.*

**Supplementary Information:**

The online version contains supplementary material available at 10.1007/s10067-024-06901-y.

## Introduction

Primary Sjögren’s syndrome (pSS) is a complex and heterogeneous autoimmune disease that leads to secretory gland dysfunction. It causes dryness of the main mucosal surfaces such as the mouth, eyes, nose, pharynx, larynx, and vagina, mainly characterized by sicca symptoms (xerostomia and xerophthalmia) [1], which can have a major impact on quality of life, including dry eye, reduced salivary flow rates, an increased risk of dental caries, and oral candidiasis [2, 3]. Approximately 20–40% of patients with pSS may experience extraglandular involvement [4], and among them lymphoma is the leading cause of death [5]. In the 2016 American College of Rheumatology (ACR)/European League Against Rheumatism (EULAR) classification criteria, serological (Anti-Ro/SSA) and histological examinations (labial salivary gland biopsy) were assigned the highest specificity and highest values [6]. However, anti-SSA antibodies can be indicative of a more advanced stage of the disease, and relying on them alone for diagnosis may result in inadequate recognition of very early pSS [7]. Labial salivary gland biopsy is an invasive examination, which may possibly cause discomfort and complications [8], and also may be affected by the subjective judgment of specimen observers [9]. Therefore, it is necessary to develop sensitive and specific biomarkers to assist the early diagnosis of pSS.

Host inflammatory responses are essential for the development and progression of pSS and are regulated by various signaling pathways, such as pro-inflammatory cytokines and interferon [10, 11], and the use of anti-inflammatory treatments has been reported to provide relief from symptoms associated with pSS [12]. At present, local tear and saliva substitutes, systemic secretagogues and immunosuppressants (glucocorticoids, chloroquine/hydroxychloroquine chloroquine and methotrexate) are commonly used treatments for pSS; however, their effectiveness is rarely seen in practice [13–15]. Targeted treatment for pSS is still unavailable despite continued research into the disease’s pathogenesis, which may be due to the lack of systematic research on targeted biomarkers. Previous studies have uncovered a substantial amount of differentially expressed genes in the SS peripheral blood sample dataset [16]. Our study sought to investigate targeted inflammation-associated biomarkers through multiple bioinformatics pathways.

Given the limitations of anti-SSA antibody detection (delay and non-specificity) and labial salivary gland biopsy (invasiveness and subjectivity) in early pSS diagnosis, regulating the inflammatory response and employing anti-inflammatory therapy have emerged as crucial management strategies for pSS [10–12]. Further, considering sensitivity and specificity of biomarker detection in serum, saliva, tears, or urine can potentially provide a more prompt and accurate reflection of the disease’s presence and progression, it has the potential to enhance the diagnostic accuracy of pSS and offer improved prospects for the treatment and intervention in early pSS. Hence, in this study, the inflammation-associated biomarkers with diagnostic value for pSS were filtered through two classical machine learning algorithms. The diagnostic value for the biomarkers was confirmed, and the biomarkers-related underlying mechanisms in pSS were initially investigate. Relevance analysis of inflammation-associated biomarkers and immune cell infiltration were performed. Moreover, the regulatory networks targeting the biomarkers were investigated, and the biomarkers-targeted drugs were predicted. Based on the five pSS-related online datasets containing the transcriptional expression profiles of whole peripheral blood samples, we make the case that the research could provide a basis for understanding disease pathogenesis and improving clinical diagnosis and treatment.

## Materials and methods

### Datasets and gene collection

Five pSS-related datasets were downloaded from the GEO database, namely the GSE51092, GSE66795, GSE84844, GSE145065, and GSE132842 datasets. The microarray datasets of GSE51092 contained the transcriptional expression profile of whole peripheral blood samples from 32 healthy controls and 190 pSS patients and was utilized to screen inflammatory-associated biomarkers, immune infiltration analysis, and single-gene GSEA analysis. Two microarray expression profiling datasets of pSS were employed to validate the expression and diagnostic value of inflammatory-associated biomarkers, that is, GSE66795 and GSE84844. GSE66795 dataset included a transcriptional expression profile of whole peripheral blood samples from 29 healthy controls and 131 pSS patients. The GSE84844 dataset comprised the transcriptional expression profiles of whole blood samples from 30 healthy controls and 30 pSS patients. The information of age and gender of patients and controls within the three pSS-related datasets above was exhibited in Table 1. Furthermore, RNA sequencing (RNA-seq) data from GSE145065 dataset, consisting of mRNA and lncRNA expression profiles of peripheral blood monocytes from 5 healthy controls and 5 pSS patients, was used for differential lncRNA screening. The GSE132842 dataset comprising miRNA expression profiles of CD1c-expressing cDC2s isolated from peripheral blood from 6 healthy controls and 15 pSS patients was detected through TaqMan OpenArray Human MicroRNA Panel and was used for differential miRNA screening. Two-hundred inflammation-associated genes were derived from the MSigDB database by searching the inflammation-associated gene set in the Hallmark gene set with the keyword ‘Inflammatory’ (Supplementary Table 1).

**Table 1:** The clinical information in the three pSS-related datasets

	GSE51092	GSE66795	GSE84844	Overall
	(*N* = 222)	(*N* = 160)	(*N* = 60)	(*N* = 442)
Group				
Control	32 (14.4%)	29 (18.1%)	30 (50.0%)	91 (20.6%)
pSS	190 (85.6%)	131 (81.9%)	30 (50.0%)	351 (79.4%)
Age				
[20, 30]	0 (0%)	0 (0%)	7 (11.7%)	7 (1.6%)
[30, 40]	0 (0%)	0 (0%)	10 (16.7%)	10 (2.3%)
[40, 50]	0 (0%)	0 (0%)	15 (25.0%)	15 (3.4%)
[50, 60]	0 (0%)	0 (0%)	10 (16.7%)	10 (2.3%)
[60, 70]	0 (0%)	0 (0%)	12 (20.0%)	12 (2.7%)
[70, 80]	0 (0%)	0 (0%)	6 (10.0%)	6 (1.4%)
Missing	222 (100%)	160 (100%)	0 (0%)	382 (86.4%)
Gender				
Female	0 (0%)	160 (100%)	59 (98.3%)	219 (49.5%)
Male	0 (0%)	0 (0%)	1 (1.7%)	1 (0.2%)
Missing	222 (100%)	0 (0%)	0 (0%)	222 (50.2%)

### Certification of candidate genes for inflammation-associated biomarkers in pSS

The ‘limma’ package (version 3.50.0) was used to authenticate the differentially expressed genes (DEGs) between pSS samples and healthy controls in the GSE51092 dataset, defined by |log_2_FoldChange (FC) > 0.5| and *p*-value < 0.05 [17, 18]. The pSS-related genes were then filtered by Weighted Gene Co-expression Network Analysis (WGCNA) in the GSE51092 dataset. The R package ‘WGCNA’ (version 1.7–3) [19] was implemented to generate a co-expression network. The determination of the soft threshold firstly ensures that the interaction between genes conforms to the scale-free distribution to the maximum extent. Through gene adjacency calculations and assessing gene similarity, the introduction of topological overlap matrix (TOM) allows for the construction of a systematic clustering tree. Further, the dynamic tree cutting method was employed to assign genes to modules under hierarchical clustering, setting the minimum number of genes per gene module to 150. In addition, the pSS samples and healthy controls were considered as trait data for WGCNA to retrieve modules and genes associated with pSS using correlation analysis. The intersection of DEGs, pSS-related genes, and inflammation-associated genes was obtained from a Venn diagram and was incorporated into the subsequent analyses as candidate genes for inflammation-associated biomarkers in pSS.

### Functional annotation analysis

The default gene set in R package ‘clusterProfiler’ (version 4.2.1) [20] was applied as background gene set for Gene Ontology (GO) and Kyoto Encyclopedia of Genes and Genomes (KEGG) enrichment analysis of candidate genes. GO was categorized into cellular component (CC), molecular function (MF), and biological process (BP). An adjusted *p*-value < 0.05 was considered statistically significant.

### Recognition of inflammation-associated biomarkers in pSS

In the GSE51092 dataset, two machine learning methods were applied to screen for disease characteristic genes, namely least absolute shrinkage and selection operator (LASSO) [21] and support vector machines recursive feature elimination (SVM-RFE) [22]. LASSO logistic regression was performed with the R software package ‘glmnet’ (version 4.0–2), setting the parameters family as binomial and type.measure as class. The error rate with different features was measured using tenfold cross-validation. By adjusting the penalty coefficient lambda, the majority of variable coefficients are eventually forced to converge to 0. The optimal lambda value is selected when the minimal error and the strong relevant features were selected. Support vector machine (SVM) analysis was carried out using the SVM in R package ‘e1071’ (version1.7–9). The best feature subset is determined by evaluating the model’s performance as the features are progressively reduced. Specifically, the recursive feature elimination (RFE) method was deployed to obtain the importance ranking of each gene, as well as the error rate and accuracy rate of each iteration of the combination. The features are gradually reduced until achieving the highest classification accuracy and lowest error rate under the feature subset size should not exceed the predefined maximum value, and the corresponding gene was extracted as the feature gene. The genes identified by both LASSO and SVM-RFE were defined as inflammation-associated biomarkers in pSS.

### Relevance analysis of inflammation-associated biomarkers and immune cell infiltration

The relative infiltration levels of 28 types of immune cells in 32 healthy controls and 190 pSS samples in the GSE51092 dataset were profiled by the ssGSEA algorithm, which was run in the ‘GSVA’ package (version 1.38.0) [23]. Variations in the infiltration levels of different immune cells between the normal and pSS samples were estimated using a Wilcoxon test, and the results were visualized by a violin plot created by the ‘vioplot’ package (version 0.3.7). The correlations between immune cells and correlations between biomarkers and differential immune cells were assessed by the Pearson method.

### Gene set enrichment analysis based on a single gene

The ‘h.all.v6.2.sytmbols.gmt’ in the MSigDB database was extracted to act as the reference gene set, and the pSS samples in the GSE51092 dataset were assigned to high- and low-expression groups with the median as the cut-off value of each key gene for calculating the fold change of gene expression between the high and low expression groups and ranking them. Furthermore, GSEA was performed to investigate the differences in gene set enrichment between the high and low expression groups using the R software ‘clusterProfiler’ (version 4.0.5) package. Significance thresholds of single gene GSEA were |NES|> 1, *q* value < 0.2, and *p* value < 0.05.

### Establishment of lncRNA-miRNA-mRNA network and drug-gene network

The miRNAs targeting the inflammation-associated biomarkers and the lncRNAs targeting miRNAs were predicted by the StarBase database (screening condition: CLIP-DATA ≥ 1). Under the screening criteria of |log_2_FC > 1| and *p-*value < 0.05, the predicted miRNAs were crossed with the differentially expressed miRNAs (DE-miRNAs) between the pSS samples and healthy controls in the GSE132842 dataset. Similarly, the predicted lncRNAs were crossed with the differentially expressed lncRNAs (DE-lncRNAs) between the pSS samples and healthy controls in the GSE145065 dataset. Moreover, the drugs targeting the inflammation-associated biomarkers were forecasted in the DGIdb database. After inputting the biomarkers, drugs related to the treatment of inflammation were obtained from the extracted drug-gene interaction information. The final lncRNA-miRNA-mRNA regulatory network and gene-drug network was mapped by Cytoscape software, where each node is presented as lncRNA, miRNA, mRNA or drug, and the edge is presented as the interaction between them in a visual way (version 3.8.2) [24].

### RNA acquisition and real-time quantitative PCR (RT-qPCR)

A total of 10 pSS patients and 10 healthy control patients were recruited from the Peking University Third Hospital with PBMC samples to perform RT-qPCR experiments. The pSS patients fulfilled the 2016 American College of Rheumatology (ACR)/European League Against Rheumatism (EULAR) classification criteria [6]. The detailed clinical information of the patients involved is shown in Supplementary Table 2. This study was approved by the Peking University Third Hospital Medical Science Research Ethics Committee (IRB00006761-M2022106), written informed consent was received from all participants for their enrollment, and all methods were carried out in accordance with relevant guidelines and regulations. The total RNA of PBMC samples from 10 healthy control and 10 pSS patients was isolated by the TRIzol Reagent following the manufacturer’s guidance (Ambion, USA). Next, total RNA was inversely transcribed into cDNA utilizing the SweScript-First-strand-cDNA-synthesis-kit (Servicebio, China), according to the manufacturer’s protocol. qPCR was subsequently performed using the 2xUniversal Blue SYBR Green qPCR Master Mix (Servicebio, China). The primer sequences for PCR are displayed in Table 2. The relative expression level was uniformized to the internal reference GAPDH and calculated using the 2^−ΔΔCq^ method [25].

**Table 2:** The primer sequences for RT-qPCR

Primer	Sequence
LY6E For	CTGTACTGCCTGAAGCCGA
LY6E Rev	CCATGGAAGCCACACCAAC
EIF2AK2 For	GCCGCTAAACTTGCATATCTTCA
EIF2AK2 Rev	TCACACGTAGTAGCAAAAGAACC
IL15 For	ATGAAGTGCTTTCTCTTGGAGT
IL15 Rev	GAAGTGTTGATGAACATTTGGA
CXCL10 For	TTCTGATTTGCTGCCTTATCTTTC
CXCL10 Rev	CTTCTCACCCTTCTTTTTCATTGT
GAPDH For	GGAAGGTGAAGGTCGGAGT
GAPDH Rev	TGAGGTCAATGAAGGGGTC

### Statistical analysis

Violin plots of gene expression were produced by the R package ‘ggstatsplot’ (version 0.9.1). ROC curves were generated by the ‘pROC’ package (1.17.0.1). All analyses were conducted using the R programming language, and the data from different groups were compared by the Wilcoxon test. The Student’s *t*-test was utilized to filter for DE-miRNAs and compare the differences in RT-qPCR. If not specified above, a *p*-value less than 0.05 was considered statistically significant.

## Results

### Candidate genes for inflammation-associated biomarkers in pSS

**Fig. 1:**
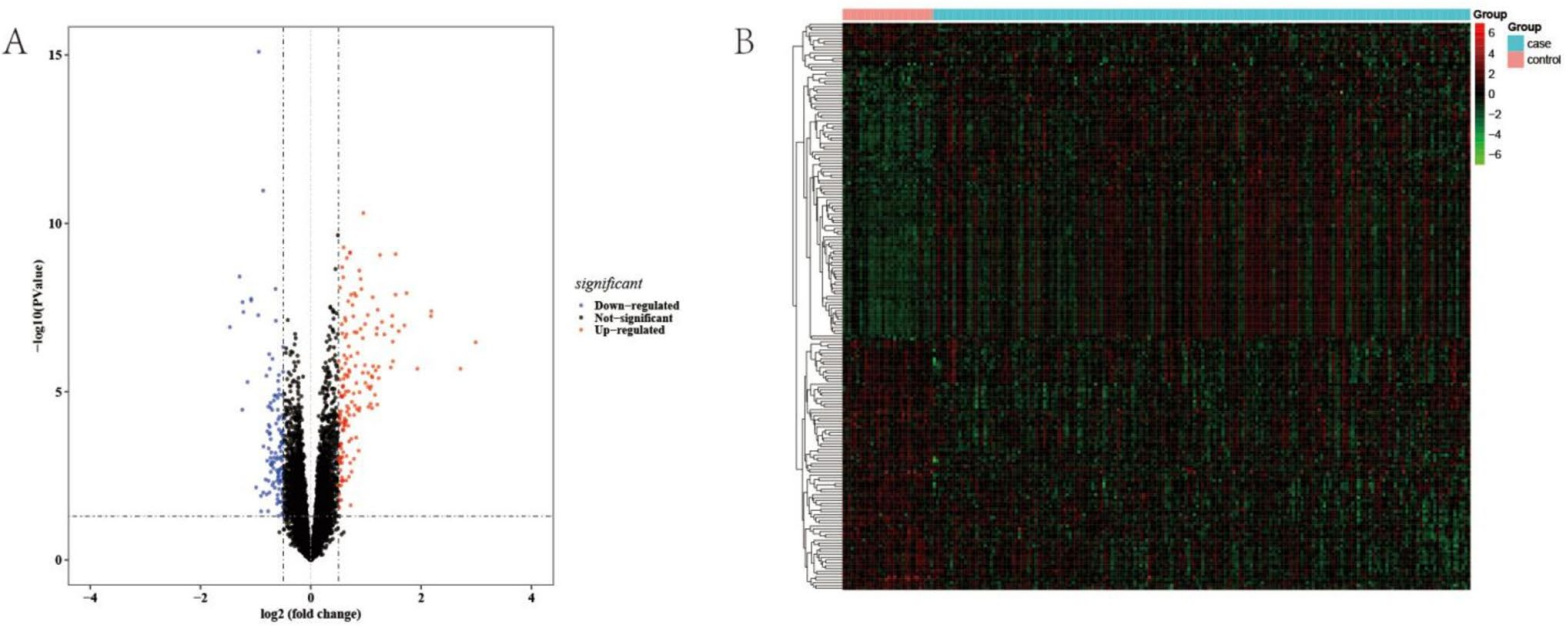
Identification of DEGs in pSS. **A** The Volcano plot of DEGs between pSS and healthy controls in the GSE51092 dataset, including 165 upregulated and 117 down-regulated genes (|log2FC|> 0.5 and *p-*value < 0.05). The top 50 DEGs were shown in the Volcano plot. **B** The heatmap of top 50 DEGs between pSS and healthy controls in the GSE51092 dataset

**Fig. 2:**
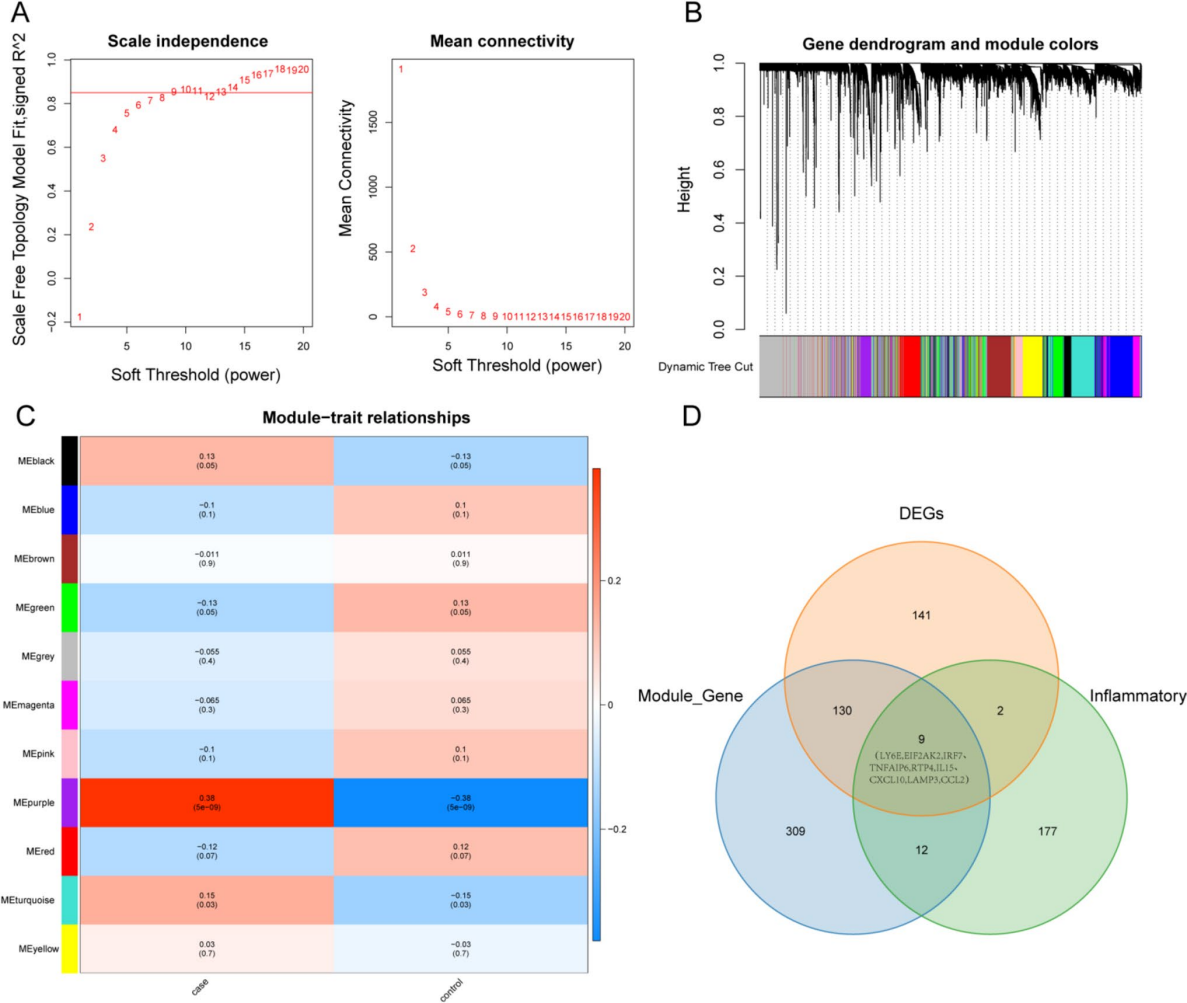
WGCNA in GSE51092 dataset. **A** Determination of soft-thresholding powers (β), including the scale-free fit index for various soft-thresholding powers (left) and the mean connectivity for various soft-thresholding powers (right). **B** Dendrogram of all DEGs clustered based on a dissimilarity measure (1-TOM) and 11 modules were displayed with corresponding colors. **C** The correlation between modules and clinical traits (control or pSS), indicating the purple module was highest correlated to clinical traits (cor = 0.38, *p* = 5e − 09). The number in the middle of each box represents the correlation coefficient, with the corresponding *p*-value in brackets. (D) Venn diagram of 9 intersected genes by overlapping DEGs, pSS-related genes identified by WGCNA, and inflammation-associated genes

The workflow diagram for the current study was displayed in Supplementary Fig. 1. To determine the differentially expressed pSS-related genes, the DEGs between pSS and healthy controls in the GSE51092 dataset were first authenticated. According to |log_2_FC|> 0.5 and *p*-value < 0.05, a grand total of 282 DEGs, including 165 upregulated and 117 downregulated genes were identified in the pSS samples (Fig. 1A–B, Supplementary Table 3). Then, WGCNA was implemented using the data of the GSE51092 dataset. Firstly, no outlier samples were excluded by sample cluster analysis (Supplementary Fig. 2A). Nine were chosen as the optimal soft threshold (*R*^2^ = 0.85) to ensure that the interactions between genes maximally conform to the scale-free distribution (Fig. 2A). Next, a total of 11 modules were developed based on a gene clustering tree and dynamic tree cutting algorithm (Fig. 2B, Supplementary Fig. 2B). Correlations between modules and sample traits (healthy control or disease pSS) were computed, and the purple module with the highest correlation was selected as the key module (Fig. 2C). Hence, the 459 genes in the key module were regarded as pSS-related genes (Supplementary Table 4). Subsequently, the DEGs, pSS-related genes, and inflammation-associated genes were overlapped, resulting in nine intersecting genes (Fig. 2D), namely LY6E, EIF2AK2, IRF7, TNFAIP6, RTP4, IL15, CXCL10, LAMP3, and CCL2. These genes were considered the candidate genes for inflammation-associated biomarkers in pSS.

**Fig. 3:**
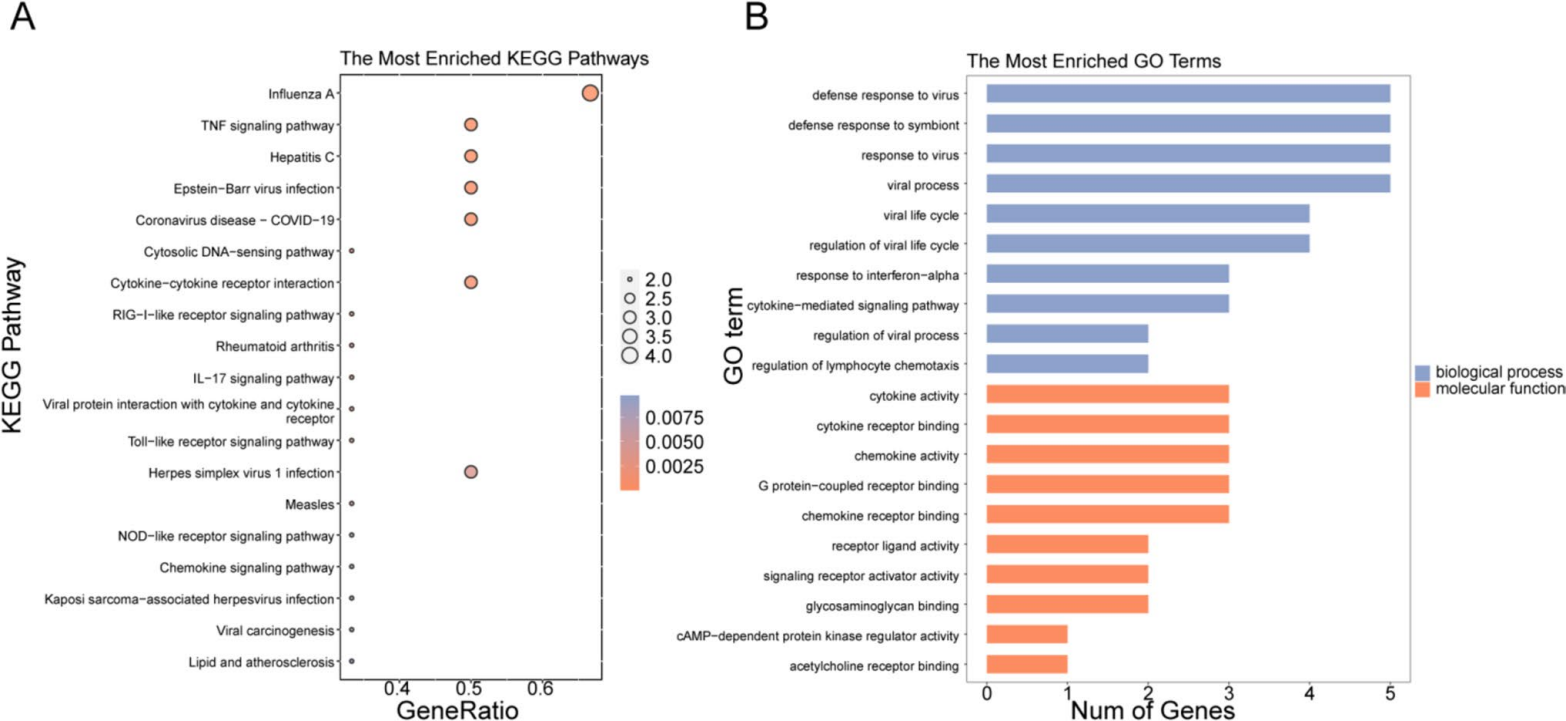
Functional analysis of candidate genes for inflammation-associated biomarkers in pSS. **A** Bubble plot for the KEGG pathways enriched by the nine candidated genes, including ‘Influenza A’, ‘TNF signaling pathway’, ‘Hepatitis C’. **B** Bar chart for the GO terms activated by the nine candidated genes, including the enriched biological processes of response to virus and the molecular functions of ‘cytokine- cytokine receptor interaction’

To further investigate the function of these nine genes, a functional enrichment analysis was executed. As displayed in Supplementary Table 5, 180 GO items (162 BP items and 18 MF items) and 19 KEGG pathways (Fig. 3A) were derived. The top 10 GO items under each classification were displayed in a bar chart (Fig. 3B). These genes were involved in many immune-related biological processes and pathways, including ‘response to interferon-alpha’, ‘cytokine-mediated signaling pathway’, ‘regulation of lymphocyte migration’, ‘cytokine-cytokine receptor interaction’, ‘TNF signaling pathway’, ‘RIG-I-like receptor signaling pathway’, ‘Toll-like receptor signaling pathway’, ‘NOD-like receptor signaling pathway’, ‘IL-17 signaling pathway’, and ‘chemokine signaling pathway’.

### Inflammation-associated biomarkers in pSS

**Fig. 4:**
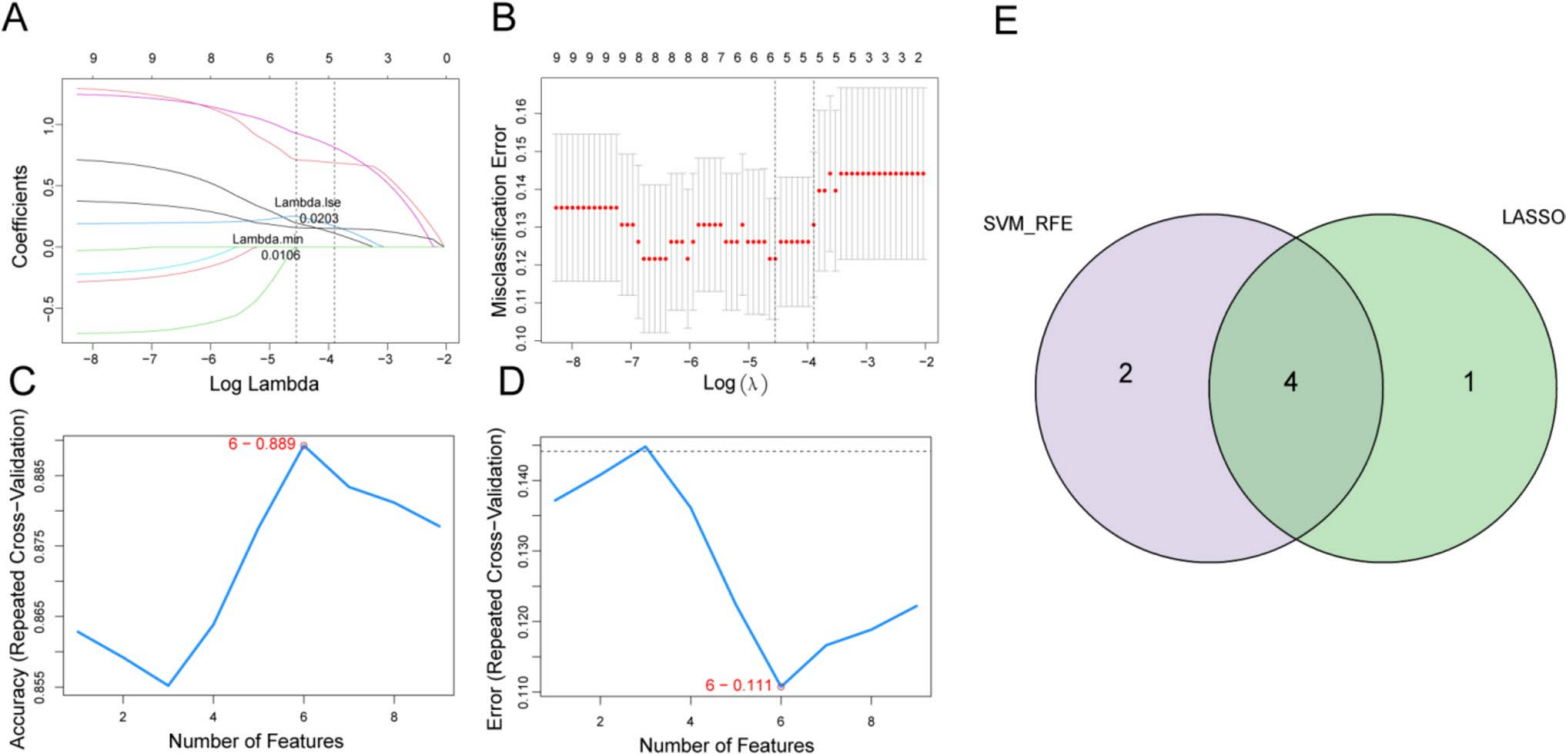
Identification of inflammation-associated biomarkers in pSS. **A** The logic coefficient penalty diagram of LASSO. **B** The cross-validation error profile of LASSO. **C** Determination of number of feature genes by the accuracy of SVM-RFE model. **D** Determination of number of feature genes by the error of SVM-RFE model. **E** Venn diagram for four overlapping inflammation-associated biomarkers

**Fig. 5:**
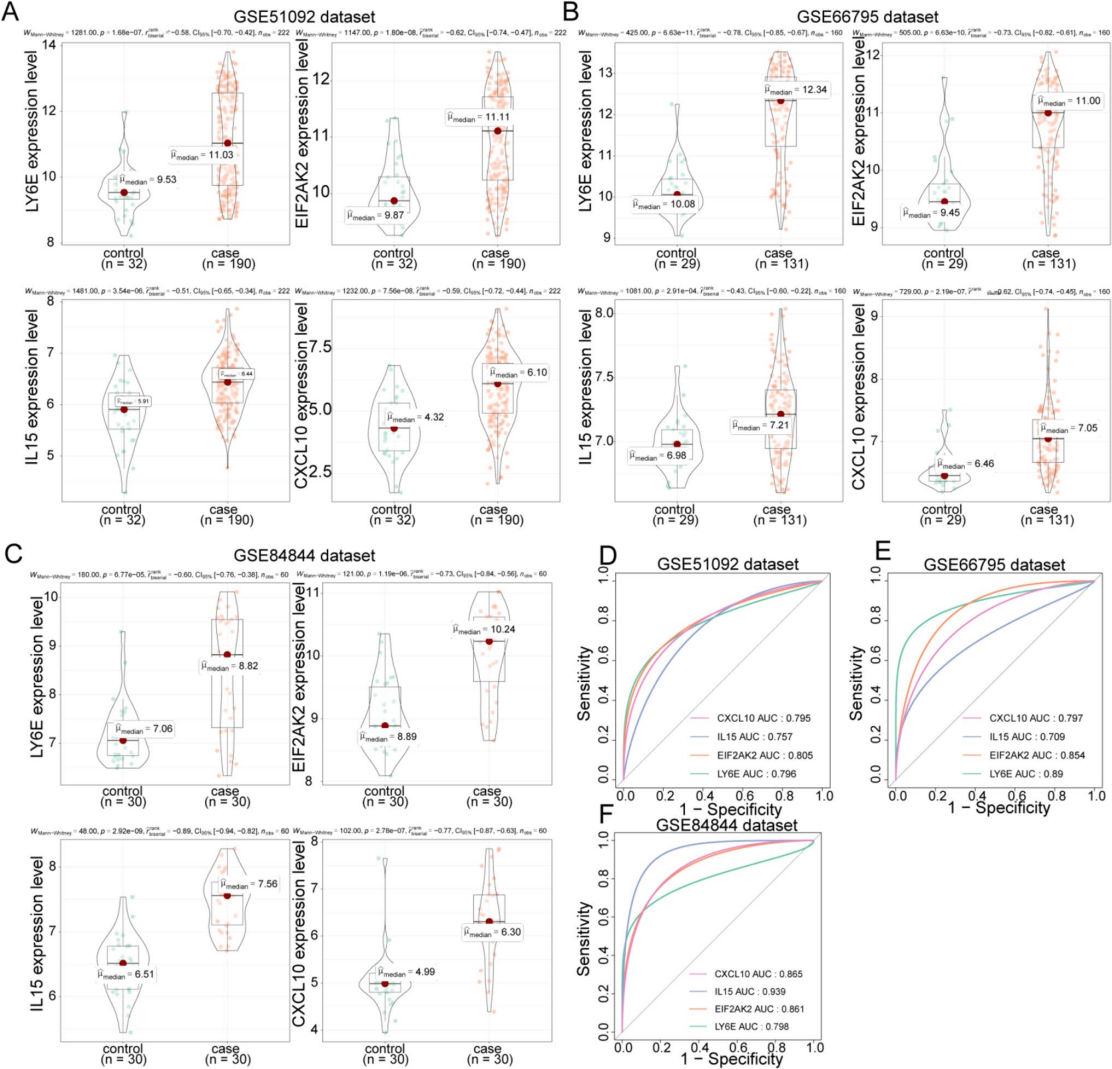
The potential diagnostic value of the inflammation-associated biomarkers in pSS. **A** The expression of inflammation-associated biomarkers in the GSE51092 dataset. **B** The expression of inflammation-associated biomarkers in the GSE66795 dataset. **C** The expression of inflammation-associated biomarkers in the GSE84844 dataset. **D** ROC curves of the four inflammation-associated biomarkers in the GSE51092 dataset. **E** ROC curves of the four inflammation-associated biomarkers in the GSE66795 dataset. **F** ROC curves of the four inflammation-associated biomarkers s in the GSE84844 dataset

To further recognize inflammation-associated biomarkers in pSS, a machine learning analysis based on the nine candidate genes was performed. As shown in Fig. 4A–B, the lowest error rate was reached at lambda.min of 0.0038, and five genes (LY6E, EIF2AK2, TNFAIP6, IL15, and CXCL10) were identified by LASSO logistic regression. Meanwhile, six genes (IL15, CXCL10, EIF2AK2, IRF7, LY6E, and CCL2) were selected by the SVM-RFE model (Fig. 4C–D). Hence, four overlapping genes (LY6E, EIF2AK2, IL15, and CXCL10) were obtained by comparing the genes obtained by the two machine learning methods (Fig. 4E). In the GSE51092 dataset, all four genes were expressed at increased levels in pSS samples compared to healthy controls (Fig. 5A). The ROC curves of the four genes in the GSE51092 dataset were mapped to further estimate the potential diagnostic value of the genes. As the AUC values all exceeded 0.7 for each gene, we concluded that the expression of each gene could effectively distinguish pSS samples from healthy controls (Fig. 5D). Furthermore, the expression of the four genes was examined, and the corresponding ROC curves for the GSE66795 and GSE84844 datasets were created to validate the above results. In agreement with the results of the GSE51092 dataset, the four genes were upregulated in the pSS samples compared to the healthy controls (Fig. 5B–C). Also, the AUC values of the ROC curves were all greater than 0.7, indicating that these four genes were reliable potential diagnostic biomarkers (Fig. 5E–F). Therefore, these four genes were defined as inflammation-associated biomarkers in pSS. To gain a preliminary understanding of the functions of which, the Panther classification system (http://pantherdb.org/) was used to annotate the four biomarkers by GO and Pathway function. The annotated GO items and pathways are shown in Supplementary Fig. 3, indicating that the four biomarkers were linked to ‘inflammation mediated by chemokine and cytokine signaling pathway’, ‘interleukin signaling pathway’, and ‘apoptosis signaling pathway’.

**Fig. 6:**
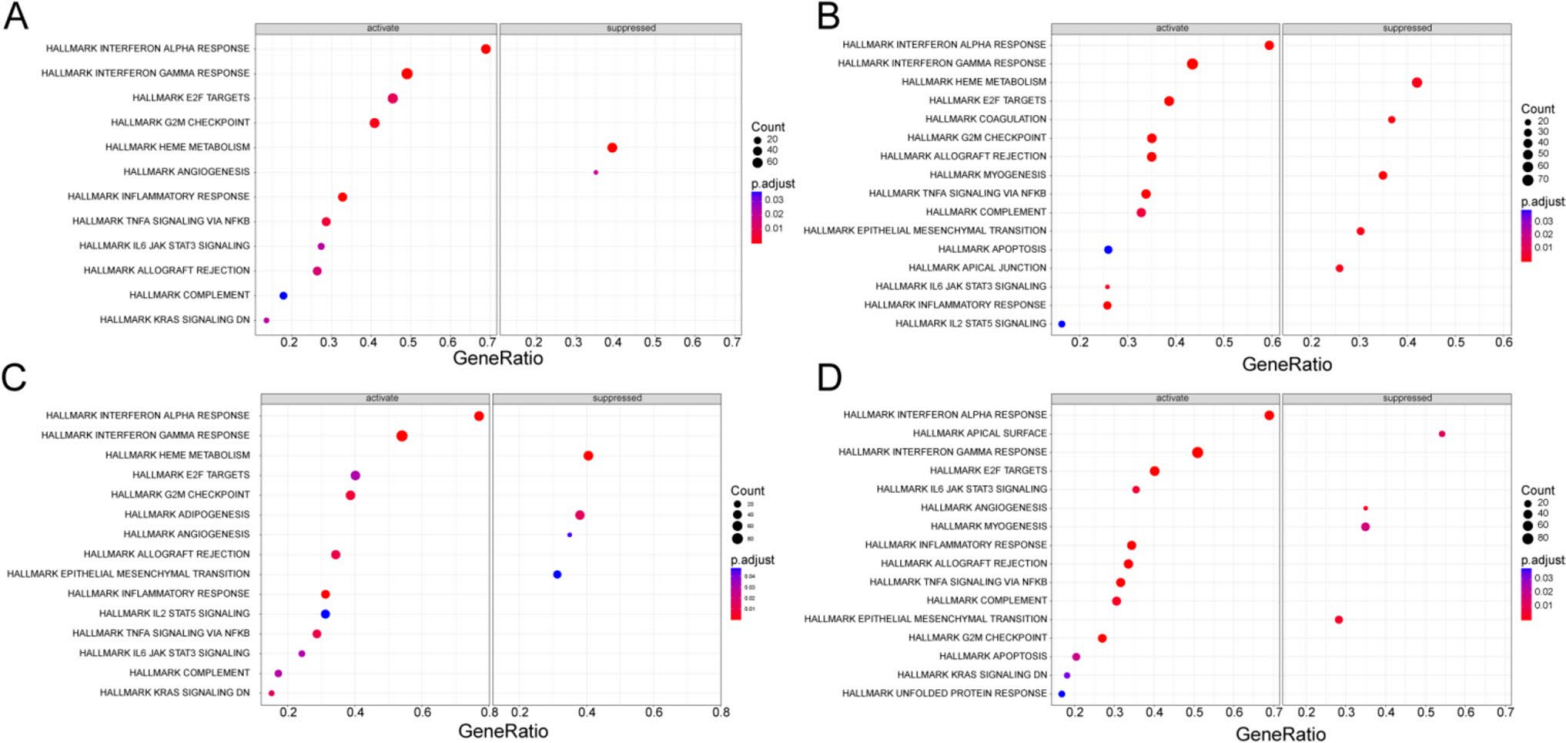
Bubble plot of single-gene GSEA enrichment analysis of four inflammation-associated biomarkers in pSS. **A** Enrichment results of LY6E by single-gene GSEA illustrated that 10 terms were activated, and 2 were suppressed based on hallmark gene set. **B** Enrichment results of EIF2AK2 by single-gene GSEA illustrated that 11 terms were activated, and 4 were suppressed based on hallmark gene set. **C** Enrichment results of IL15 by single-gene GSEA illustrated that 11 terms were activated, and 5 were suppressed based on hallmark gene set. **D** Enrichment results of CXCL10 by single-gene GSEA illustrated that 12 terms were activated, and 4 were suppressed based on hallmark gene set

Subsequently, the single-gene GSEA was performed based on the hallmark gene set and transcriptomic data of pSS samples in the GSE51092 dataset to explore the molecular mechanisms of each inflammation-associated biomarker in pSS. As revealed in Fig. 6, all four genes were linked to the activation of ‘HALLMARK_INTERFERON_GAMMA_RESPONSE’, and ‘HALLMARK_INTERFERON_ALPHA_RESPONSE’, in the meantime, the inhibition of ‘HALLMARK HEME METABOLISM’ should be possible to related to EIF2AK2, IL15 and LY6E. More details could be found in the Supplementary Table 6.

### The relevance of inflammation-associated biomarkers to immune cells in pSS

**Fig. 7:**
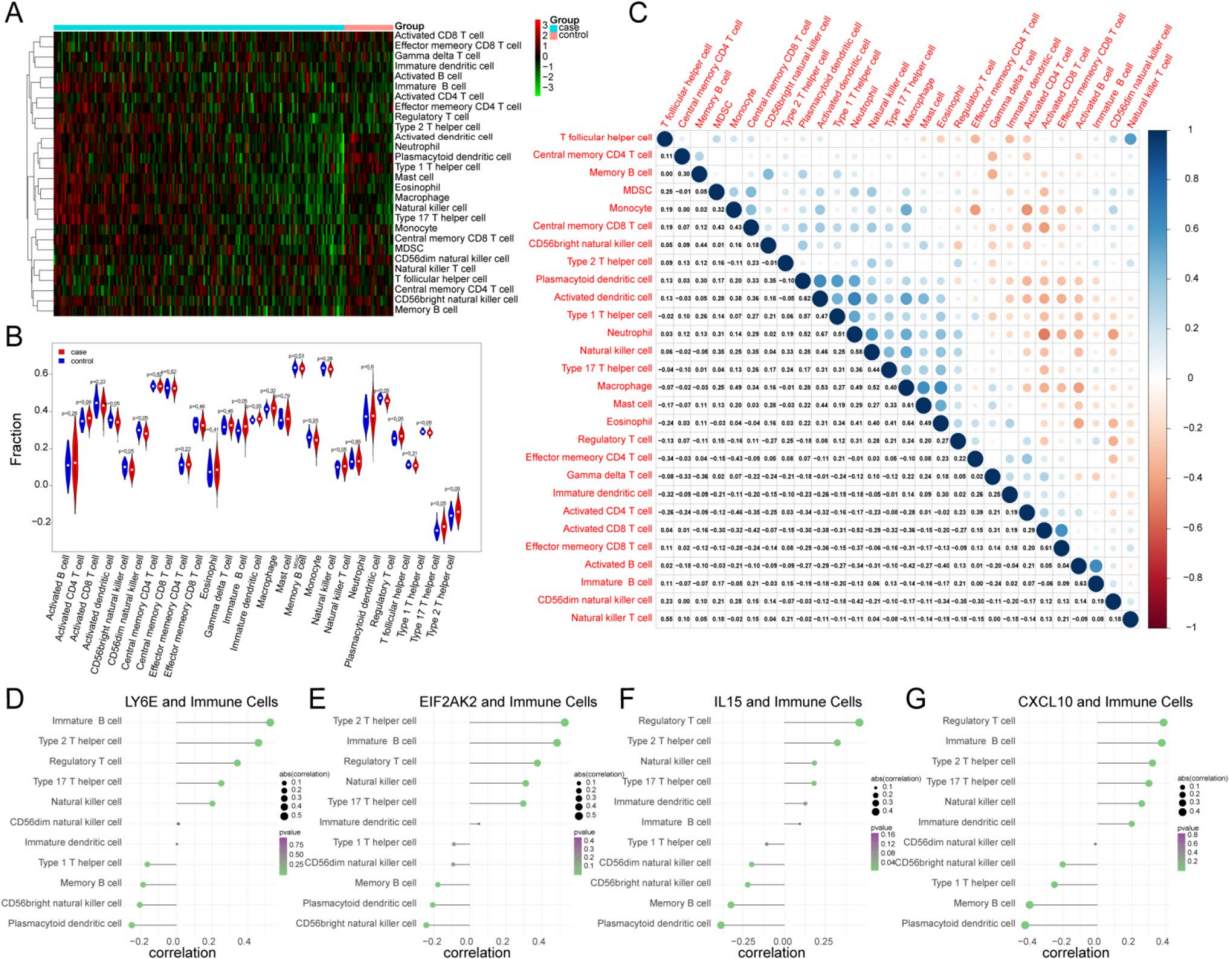
The relevance of inflammation-associated biomarkers to immune cells in pSS. **A** The heatmap of ssGSEA scores of immune cells infiltration for each sample in the GSE51092 dataset. **B** The violin plot of comparing infiltration levels of immune cells between pSS and control samples. **C** The correlation between different immune cells. (D) Pearson correlation of LY6E and the immune cells. **E** Pearson correlation of EIF2AK2 and the immune cells. (F) Pearson correlation of IL15 and the immune cells. **G** Pearson correlation of CXCL10 and the immune cells. **B** Difference in the infiltration levels of immune cells between pSS and control samples were compared using wilcox.test. **C** The correlation heatmap among different immune cells was displayed through Pearson correlation analysis. **D** Pearson correlation results showed that LY6E was significantly correlated to Immature B cells, Regulatory T cells and Type 2 T helper cells. **E** Pearson correlation results showed that EIF2AK2 was closely relevent to Immature B cells, Natural killer cells, Regulatory T cells and Type 2 T helper cells. **F** Pearson correlation results showed that IL15 was negatively correlated to Memory B cells, Plasmacytoid dendritic cells while it was positively correlated to Regulatory T cells and Type 2 T helper cells. **G** Pearson correlation results showed that CXCL10 was negatively correlated to Memory B cells, Plasmacytoid dendritic cells as well, while it was positively correlated to Immature B cells, Regulatory T cells and Type 2 T helper cells

To further probe the relationship between inflammation-associated biomarkers and immune cells in pSS, the infiltration level of 28 types of immune cells between pSS samples and healthy controls in the GSE51092 dataset were first compared through the ssGSEA method. The scores for each immune infiltrating cell in each sample are shown in the heatmap (Fig. 7A). The violin plot suggested that the enrichment fraction of CD56 bright natural killer cells, CD56 dim natural killer cells, plasmacytoid dendritic cells, memory B cells, type 1 T helper cells, immature B cells, immature dendritic cells, natural killer cells, regulatory T cells, type 17 T helper cells, and type 2 T helper cells had significant differences between healthy controls and pSS samples (Fig. 7B), moreover, the plasmacytoid dendritic cells were closely relevant to type 1 T helper cells (cor = 0.54) (Fig. 7C). Then, the Pearson method was employed to compute the correlation between biomarkers and differential immune cells. As illustrated in Supplementary Table 7 and Fig. 7D–G, all four biomarkers were notably positively correlated with regulatory T cells and type 2 T helper cells as defined by |correlation coefficient|> 0.3 and *p* value < 0.05. Meanwhile, CXCL10, EIF2AK2 and LY6E were prominently positively correlated with immature B cells, while IL15 were significantly negatively correlated with memory B cells and plasmacytoid dendritic cells, which were consistent with CXCL10 as well.

### The lncRNA-miRNA-mRNA network and gene-drug network based on inflammation-associated biomarkers in pSS

**Fig. 8:**
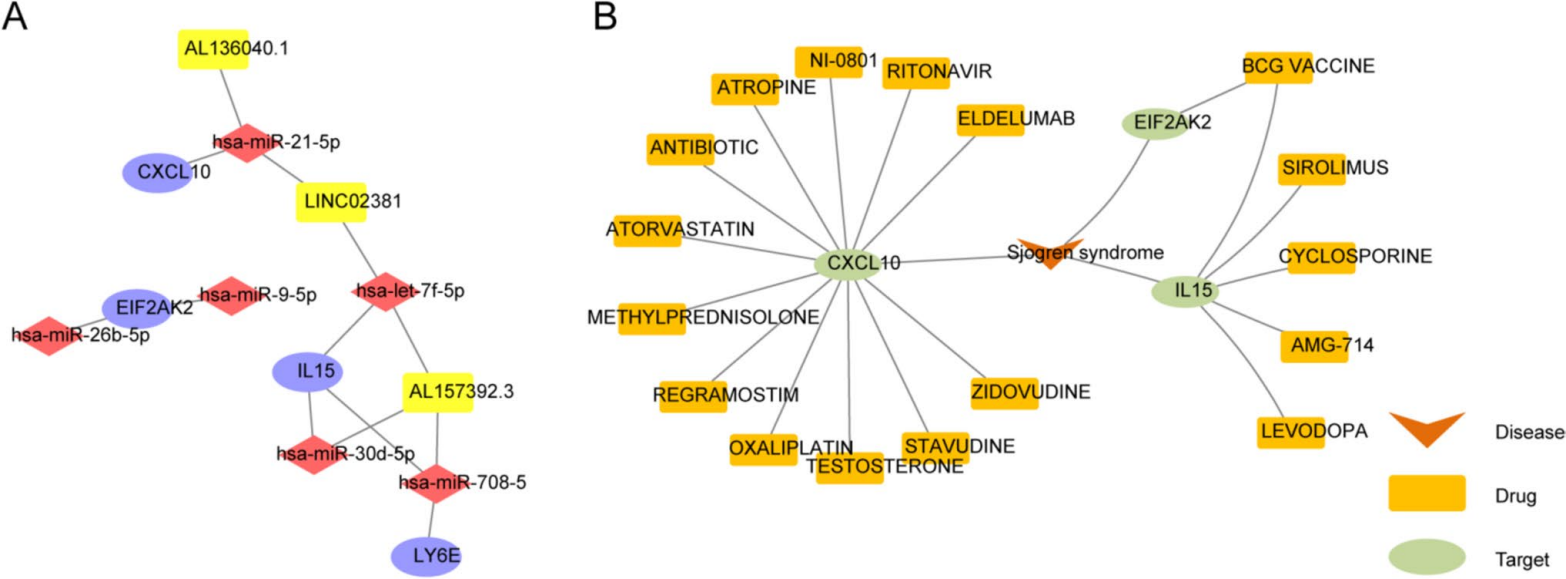
The competing endogenous RNA (ceRNA) network and the disease-genes-drugs network relevant to the inflammation-associated biomarkers. **A** The lncRNA-miRNA-mRNA network targeting inflammation-associated biomarkers. Yellow rectangle represents lncRNA, red diamond represents miRNA, and purple oval represents mRNA. **B** The potential disease-gene-drug network of inflammation-associated biomarkers through DGIdb database, while the potential drug targeting the LY6E gene are lacking

The DE-miRNAs between the pSS and healthy controls in the GSE132842 dataset were screened to investigate the upstream regulatory mechanisms of inflammation-associated biomarkers. A total of 12 DE-miRNAs were mined according to the screening conditions *p* value < 0.05 and |log_2_FC|> 1, of which 2 were highly expressed, and 10 showed low expression in pSS samples (Supplementary Fig. 4A–B). Meanwhile, 311 miRNAs targeting inflammation-associated biomarkers were predicted from the StarBase database. Six target miRNAs were obtained by intersecting with the 12 DE-miRNAs (Supplementary Fig. 4C). Next, the DE-lncRNAs between pSS and healthy controls in the GSE145065 dataset were screened. 63 DE-lncRNAs were identified based on the screening criteria *p*-value < 0.05 and |log_2_FC|> 1, of which 37 were highly expressed and 26 were lowly expressed in pSS (Supplementary Fig. 4D–E). Furthermore, 197 lncRNAs targeting the target miRNAs were predicted from the StarBase database. Then, 3 target lncRNAs were obtained by intersecting with the 63 DE-lncRNAs (Supplementary Fig. 4F). Finally, a lncRNA-miRNA-mRNA network with 13 nodes and 13 edges was generated using Cytoscape (Fig. 8A). In this network, hsa-miR-26-5p and hsa-miR-9-5p regulated EIF2AK2. CXCL10 was regulated by hsa-miR-21-5p, which was regulated by AL136040.1 and LINC02381. Moreover, LY6E was regulated by hsa-miR-708-5p, which was regulated by AL157392.3. IL15 was regulated by hsa-miR-30d-5p, hsa-miR-708-5p, and hsa-let-7f-5p, which were regulated by AL157392.3. In addition, hsa-let-7f-5p was regulated by LINC02381.

To explore potential drugs targeting the four inflammation-associated markers, 17 drugs targeting three biomarkers were predicted through the DGIdb database. Ultimately, a disease-gene-drug network containing 21 nodes and 21 edges was constructed (Fig. 8B). In this network, CYCLOSPORINE, SIROLIMUS and AMG714 might be associated with IL15. Furthermore, 12 drugs (ATORVASTATIN, METHYLPREDNISOLONE, TESTOSTERONE, etc.) were predicted to target CXCL10.

### Verification of the expression of inflammation-associated biomarkers in clinical samples

**Fig. 9:**
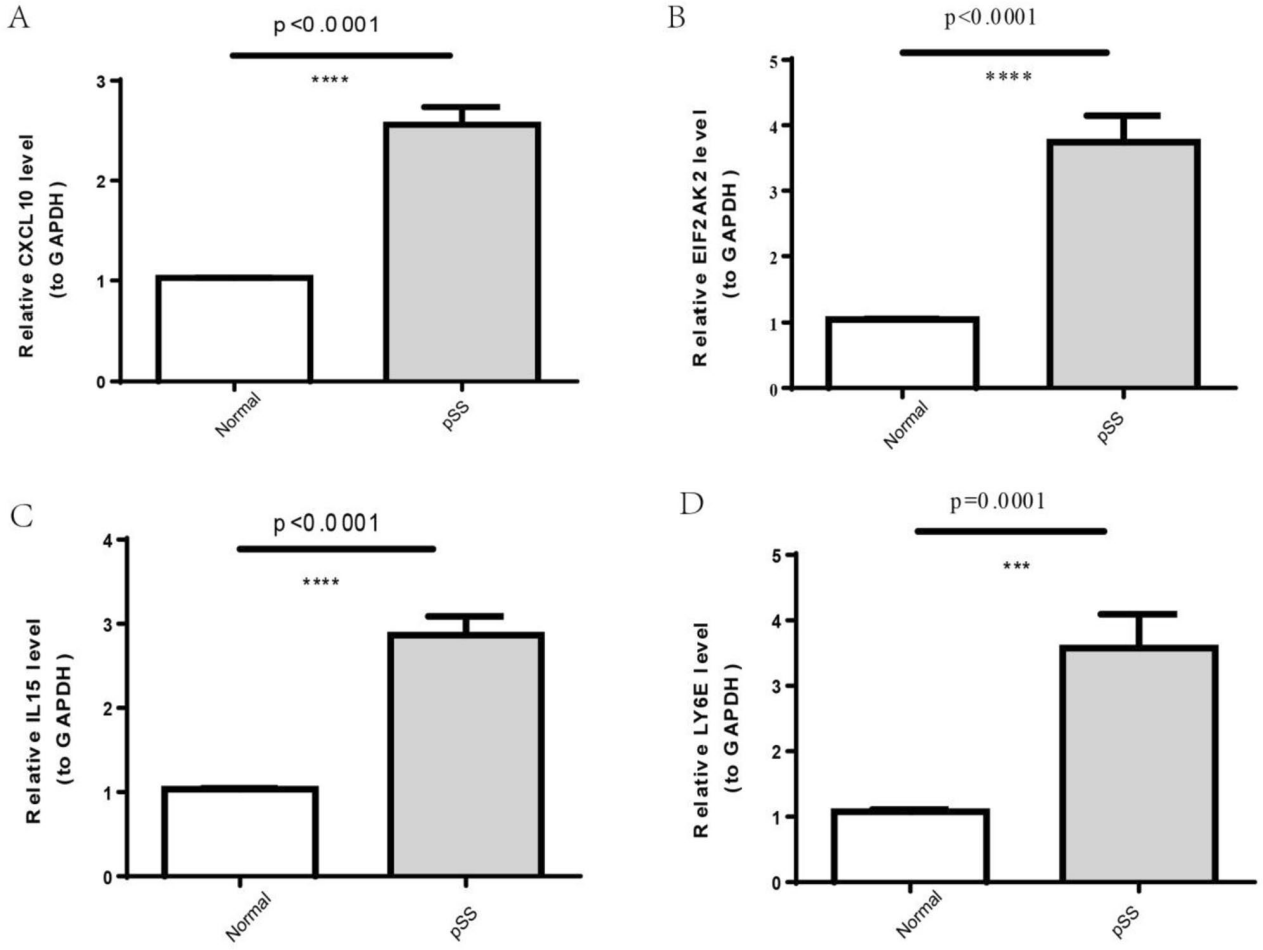
The expression levels of inflammation-associated biomarkers in pSS and healthy control subgroups patients (*n* = 10) were detected by RT-qPCR through Student’s *t*-test, indicating the over-expression of four biomarkers in pSS compared with healthy control subgroups patients. **A** CXCL10. **B** EIF2AK2. **C** IL15. **D** LY6E. * *p* < 0.05, ** *p* < 0.01, *** *p* < 0.001

As illustrated in Fig. 5A–C, all four inflammation-associated biomarkers were upregulated in pSS samples compared to healthy controls. The expression in clinical PBMC samples from 10 healthy controls and 10 pSS patients was further confirmed by RT-qPCR. In agreement with the results of the analysis of public RNA-sequencing data, four biomarkers were significantly more highly expressed in clinical pSS samples compared to healthy control samples (Fig. 9, Table 3).

**Table 3:** The statistic results of four inflammation-associated biomarkers in clinical samples

	N	pSS	FC	t, df value	*p* value
LY6E	1.0785 ± 0.1054	3.5786 ± 1.6249	3.318127028	t = 4.855 df = 18	0.0001
EIF2AK2	1.0471 ± 0.0298	3.7453 ± 1.2776	3.576831248	t = 2.266 df = 9	< 0.0001
IL15	1.0352 ± 0.0329	2.8646 ± 0.7050	2.767194745	t = 8.196 df = 9	< 0.0001
CXCL10	1.0263 ± 0.0110	2.5598 ± 0.5605	2.494202475	t = 8.622 df = 9	< 0.0001

*FC*, fold change

## Discussion

pSS is characterized by chronic inflammation and is manifested by impaired function of the exocrine glands, and mononuclear cells infiltrate surrounding the ducts and replacing the secretory units of the involved glands [26]. Due to the heterogeneities of clinical phenotypes and various causes, the identification of key biomarkers in pSS is critical to understanding the pathogenesis of this complex disease. Considering the biological significance of inflammatory response in pSS progress, the study was utilized to identify the potential inflammation-associated biomarkers through the bioinformatics methods based on the online datasets of pSS.

Using the differentially expressed analysis between pSS samples and healthy controls, as well as WCGNA in the GSE51092 datasets, nine pSS-related and inflammation-associated DEGs were identified in the present study, namely LY6E, EIF2AK2, IRF7, TNFAIP6, RTP4, IL15, CXCL10, LAMP3, and CCL2, which were mainly involved in the activation of the innate antiviral immunity process and inflammatory-related signaling pathways. Evidence has indicated that viral infections alter the clinical manifestations of various autoimmune diseases. On the other hand, protective effects can be achieved by suppressing autoimmune phenomena through regulatory immune responses [27]. Influenza viruses and EBV infection were considered as central roles in the pathogenesis of pSS through the autoimmunity induced by different mechanisms in previous literature [28–33].

Several functions relevant to the pSS-related inflammation-associated DEGs were related to immune and inflammation signaling pathways. **Toll-like receptors (TLRs)** could sense nucleic acids derived from viruses and trigger antiviral innate immune responses as pattern-recognition receptors (PRRs) [34, 35], where NF-kappaB, MAPK kinases, and IRFs that control the transcription of genes encoding type I interferon and other inflammatory cytokines were activated to eliminate viruses [36]. Previous studies have shown that TLRs play an essential role in the pathogenesis of pSS [37, 38]. They are elevated in salivary tissue [39] and in the peripheral blood of pSS patients [40]. Emerging data indicate that damage-associated molecular patterns (DAMPs) may be significant drivers of chronic and unremitting inflammation in pSS, although the ligands activating TLRs in pSS remain unknown [41, 42]. Activating TLR signaling cascades likely reduce local and systemic inflammation, as shown in an animal study [43]. There is no doubt that the interaction of the Toll-like signaling pathways and the viral defense response process may be important in pSS.

**Nod-like receptor protein 3 (NLRP3)** is a crucial player in regulating host immune responses to infection and cells stress [44], and it was also found highly expressed in pSS patients than control [45]. The NLRP3 inflammasome can be triggered by the P2X7 receptor (P2X7R), leading to acute inflammatory responses. Baldini et al. proposed the P2X7R-inflammasome axis as a novel potential pathway in both pSS exocrinopathy and lymphomagenesis [46]. These results suggested that NLRP3 inflammasome-mediated inflammation might be implicated in the pathogenesis of pSS. **Interleukin-17 (IL-17)** is a multifaceted cytokine with a well-recognized role in immune surveillance at mucosal and barrier surfaces [47]. Previous research suggests that the IL-17 axis plays a pivotal role in the pathogenesis of several autoimmune disorders, including pSS [48, 49]. Studies have demonstrated that IL-17 was overexpressed in the salivary glands (SGs) [50], serum [51], plasma [52] and tears [53] of pSS patients, and IL-17 mRNA levels in MSG biopsies seemed to be related to the degree of inflammation [52, 54]. Different IL-17 family members may play several pathogenetic roles in the development of pSS. According to a recent study, IL-17F production in pSS patients is associated with a higher level of autoantibodies and EULAR SS disease activity index (ESSDAI) than IL-17A production in pSS patients [55].

Next, four genes (**LY6E, EIF2AK2, IL15, and CXCL10**) were authenticated as inflammation-associated pSS biomarkers, and the reliability of them in discriminating pSS samples from healthy control samples, suggesting a potential clinical diagnostic value. Functional enrichment results and immune infiltration analysis pointed to the involvement of the four genes in the immune process and inflammation-related pathways in pSS. **The Lymphocyte antigen 6E (LY6E) protein** belongs to the Ly6/uPAR family of plasminogen activator receptors and is known as one of the IFN type I response genes. Recent studies have reported its essential role in immunological regulation, T cells physiology, oncogenesis, and viral infection [56]. Our study found higher LY6E levels in the peripheral blood of pSS patients, which has been proven by previous clinical studies [57–59]. These findings may reveal the importance of the peripheral blood LY6E levels and the monocyte IFN type I signature in pSS patients. **The eukaryotic translation initiation factor 2-α kinase 2 (EIF2AK2)** gene is located on chromosome 2 and encodes modifying protein kinase R (PKR, interferon-induced, double-stranded RNA-activated protein kinase) [60]. Recent studies have revealed that the coding gene PKR is associated with the treatment of pSS, which further confirms the role of EIF2AK2 in the progression of pSS [39, 61, 62]. Although LY6E and EIF2AK2 have been found as pSS diagnostic genes in previous studies [57], we further investigated the potential ceRNA regulatory network and related drugs of LY6E and EIF2AK2 in the context of inflammation, providing insight into the direction for future research. **Interleukin-15 (IL-15)** is a crucial regulatory inflammatory cytokine that is upregulated in autoimmunity disorders [63, 64]. Previous studies revealed a higher IL-15 expression level in the peripheral blood of pSS patients [65], which is consistent with our results. Besides, based on gene and protein analysis and immunohistochemical results in minor salivary gland (MSG) biopsy specimens and human salivary gland epithelial cells (SGEC) obtained from patients with pSS, IL15 was documented a strong expression in acinar and duct cells of salivary glands with pSS, which may be related to TLR2/IL-15 signaling pathway [66–69]. It’s consistent with our functional enrichment results (Toll-like receptor signaling pathway), which provides a theoretical basis for the detection of pSS by blood, but the protein levels in blood need further analyses. **C-X-C motif chemokine ligand 10 (CXCL10)** protein is categorized functionally as a Th1-chemokine, and its secretion is regulated by interferon (IFN)-γ [70]. The serum and/or tissue expressions of CXCL10 in various autoimmune diseases [70–73]. A study that assessed CXCL10 plasma levels in pSS patients showed that the ratio of full-length (active) CXCL10 to truncated DPP4-truncated (inactive) CXCL10 was significantly increased in pSS patients and provided the highest correlation with disease activity [74]. Elevated CXCL10 levels were also found in the salivary gland of pSS patients, which were associated with decreased circulating CXCR3 + helper cells, suggesting facilitating their concerted migration [75]. These results guarantee the accuracy of our transcriptome analysis results.

The pathogenesis of pSS is multifactorial and complex. The process primarily encompasses antigen presentation, costimulation, B cell activation, and other related mechanisms [11], in which the cytokine profiles of Th1, Th2, Th17, follicular helper T (Tfh) cells, and regulatory cells (Tregs/Bregs) play important roles [76]. Studies have shown that the frequency of Foxp3 + regulatory T cells (Treg) in salivary glands may be correlated with glandular infiltration and the grade of local inflammation [77], while B cell activation is generally associated with an increased risk of lymphoma [78]. Lymphocytic infiltration in salivary and lacrimal glands and the deposition of autoantibodies, like anti-SS-A (anti-Ro) and anti-SS-B (anti-La), cause an autoimmune outbreak and chronic inflammation, leading to the destruction of the salivary gland architecture [79].

In this study, we found that four key genes were significantly associated with regulatory T (Treg) cells and type 2 T helper (Th2) cells via immune infiltration and Pearson correlation analysis. Treg cell deficiency has been documented in pSS patients [80], with peripheral blood levels significantly lower than those of healthy controls, suggesting that Treg cell deficiency may be involved in salivary gland destruction [81]. Type 2 immune response which Th2 cells involved in has a regulatory relationship with autoinflammation [82]. Th2 cells have been found to promote renal inflammation in patients with systemic lupus erythematosus [83], and to play a part in the process of pSS by participating in costimulation and assisting B cell activation, with the cytokines they produce dominating the early stages of pSS [84]. These findings demonstrate that significant changes occur in Treg cells and Th2 cells in pSS and other related autoimmune diseases.

The identified miRNAs in the present study exhibited consistency with other research on autoimmune or immune-mediated related diseases. The miR-26 expression level was downregulated in multiple sclerosis (MS) patients compared to controls [85]. The neuroregulatory miRNA miR-9-5p was significantly upregulated in the peripheral blood samples of HLA-B27( +) radiographic axial spondyloarthropathy (rad-AxSpA) patients [86]. Immuno-miRNAs miR-21-5p and let-7f-5p were significantly elevated in the serum of patients with acetylcholine receptor myasthenia gravis (AChR^+^-MG) [87–90], and miR-21-5p was also upregulated in type 1 autoimmune pancreatitis (AIP) [91] and psoriatic arthritis (PsA) [92]. Kim et al. found significantly downregulated expression of miR-30d-5p in the tear samples of pSS patients [93]. These miRNAs may be involved in disease pathogenesis via immune-related processes.

In our study, three lncRNAs were identified as being associated with pSS, namely AL 136040.1, LINC02381, and AL157392.3. Previous reports have suggested that these three lncRNAs may be implicated in immunological disorders. The competitive binding of LINC02381 with miR-21 has been experimentally confirmed in previous studies. Zhao et al. demonstrated this interaction through luciferase reporter gene and RNA immunoprecipitation assays, indicating that LINC02381 sponged miR-21 to enhance KLF12 expression [94]. However, the interaction between miR-21 and LINC02381/CXCL10 still requires further validation through additional functional experiments. Additionally, Jafarzadeh et al. also confirmed LINC02381 sponged miR-21 through dual luciferase assay [95]. LINC02381/hsa-let-7f-5p/IL-6 competitive network in another immune-mediated connective tissue disease systemic sclerosis (SSc) was shown to be potentially involved in inflammatory and immune processes immune microenvironmental variation [96]. Glycolysis-associated lncRNA AL157392.3 may influence immune-related signaling in pan-cancer analysis [97].

Additionally, we predicted potential drugs based on drug-gene interaction pairs, which included glucocorticoids and immunosuppressive drugs. These drugs have been successfully used to treat autoimmune diseases. AMG-714 was used to treat celiac disease [98], which is known as an associated autoimmune disease with pSS sharing a common genetic background [99]. LEVODOPA is an effective and well-tolerated drug for the treatment of Parkinson’s disease [100], which may have a potential association with pSS [101], this suggests that IL-15 may be a potential target [100]. ZIDOVUDINE for pSS has been cited in the manuscript, but studies have shown that antiretroviral therapy has a number of severe and life-threatening adverse drug reactions. For instance, taking ZIDOVUDINE was observed as a risk factor for anemia. STAVUDINE was utilized for the treatment of peripheral neuropathy, but among that, the use of nevirapine was identified as a risk factor for cutaneous reactions [102].

Significantly, CYCLOSPORINE A has been found to be a potent inhibitor of IL-15 release in the context of acute rejection following heart transplantation in mice [103]. However, varying doses of CYCLOSPORINE, which is a key immunosuppressive therapy for kidney transplant recipients, do not appear to have an impact on serum levels of IL-15 and IP-10 cytokines [104]. While certain studies have proposed a potential role of TESTOSTERONE in modulating disease progression through the promotion of anti-inflammatory responses, the observed reduction in CXCL10 levels in male patients receiving TESTOSTERONE supplementation was not notably significant [105]. IL-15 and IP-10, in conjunction with CYCLOSPORINE, have been identified as significant inflammatory biomarkers in rheumatoid arthritis [106]. Given the notable upregulation of IL-15 and CXCL10 in pSS patients, it is postulated that pSS may contribute to the regulation of these cytokine levels via alternative mechanisms. Combined with the current research on the application of TESTOSTERONE and CYCLOSPORINE in autoimmune diseases [107–109], we speculate that TESTOSTERONE and CYCLOSPORINE may regulate the abnormal activity of immune cells and reduce inflammation by targeting the inhibition of CXCL10, a proinflammatory cytokine, and IL15, an activator of immune cells. In turn, this will help improve the immune function of pSS patients and alleviate their symptoms and immune-mediated inflammation-related damage. However, it remains to be clinically verified in pSS patients.

However, there are still several limitations in our study: Verifying the reliability of transcriptional changes in gene expression establishes a theoretical foundation for the rapid evaluation of biomarkers expression in peripheral blood detection, while detecting gene expression at the protein level requires the further detection of specific proteins or cell surface markers, using techniques such as ELISA and flow cytometry. At the same time, the diagnostic efficacy of biomarkers, drug targeting results, and the regulatory networks are currently only preliminary findings from bioinformatics research and prediction, and it is necessary to conduct larger studies with a broader cohort of patients, as well as additional follow-up RNA-seq and animal studies, to validate their effectiveness, safety, and robustness. Furthermore, conducting clinical trials is necessary to verify the interaction mechanism between key genes and key immune cells using real data obtained from an increased number of clinical samples. Despite the challenges presented, the advances in genomics offer us a unique opportunity to gain a better understanding of the pathomechanism of pSS and develop novel therapeutic strategies. Further research into pSS could result in innovative treatments.

In conclusion, four genes (LY6E, EIF2AK2, IL15, CXCL10) that might be potential diagnostic inflammation-associated biomarkers of pSS in peripheral blood were identified by bioinformatics analysis, and their expression were validated by RT-qPCR. Given that the samples used in this study were all derived from peripheral blood for the pSS-datasets, we argue that leveraging peripheral blood tests for rapid evaluation of biomarker expression has the potential to improve the diagnostic accuracy of early pSS. Furthermore, the molecular mechanisms of these genes were preliminarily explored by generating a lncRNA-miRNA-mRNA regulatory network. And meanwhile, the predicted drugs, such as TESTOSTERONE targeting CXCL10 and CYCLOSPORINE targeting IL15, may potentially enhance immune function and alleviate symptoms and immune-mediated inflammation-related damage in patients with pSS. The results provided a basis for understanding the pathogenesis and improving clinical diagnosis and treatment for pSS.

### Supplementary Information

Below is the link to the electronic supplementary material.

Supplementary file1 (TIF 1521 KB)

Supplementary file2 (TIF 14098 KB)

Supplementary file3 (TIF 21525 KB)

Supplementary file4 (TIF 13542 KB)

Supplementary file5 (XLSX 12 KB)

Supplementary file6 (XLSX 13 KB)

Supplementary file7 (XLSX 40 KB)

Supplementary file8 (XLSX 15 KB)

Supplementary file9 (XLSX 28 KB)

Supplementary file10 (XLSX 24 KB)

Supplementary file11 (XLSX 12 KB)

## Data Availability

The datasets (GSE51092, GSE66795, GSE84844, GSE145065, and GSE132842 datasets) generated and/or analysed during the current study are available in the GEO repository (https://www.ncbi.nlm.nih.gov/geo/).
