# Supplementary figures and images for: Identification and verification of inflammatory biomarkers for primary Sjögren’s syndrome
Source: Clin Rheumatol. 2024 Feb 20;43(4):1335–52. doi: 10.1007/s10067-024-06901-y (PMC10944815; doi:10.1007/s10067-024-06901-y)

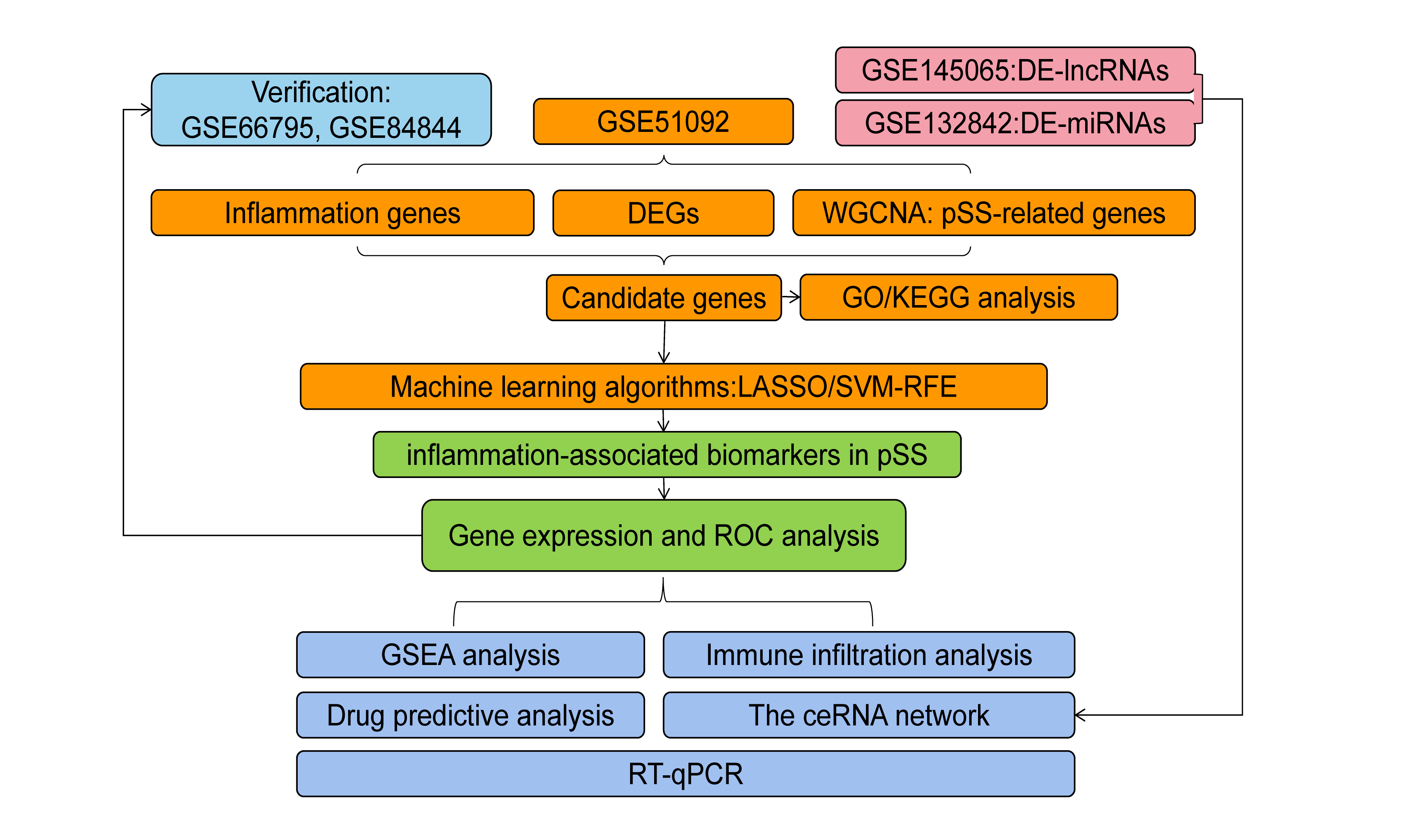

Supplement: Supplementary file 1 — Supplementary file1 (TIF 1521 KB) [file 10067_2024_6901_MOESM1_ESM.tif]

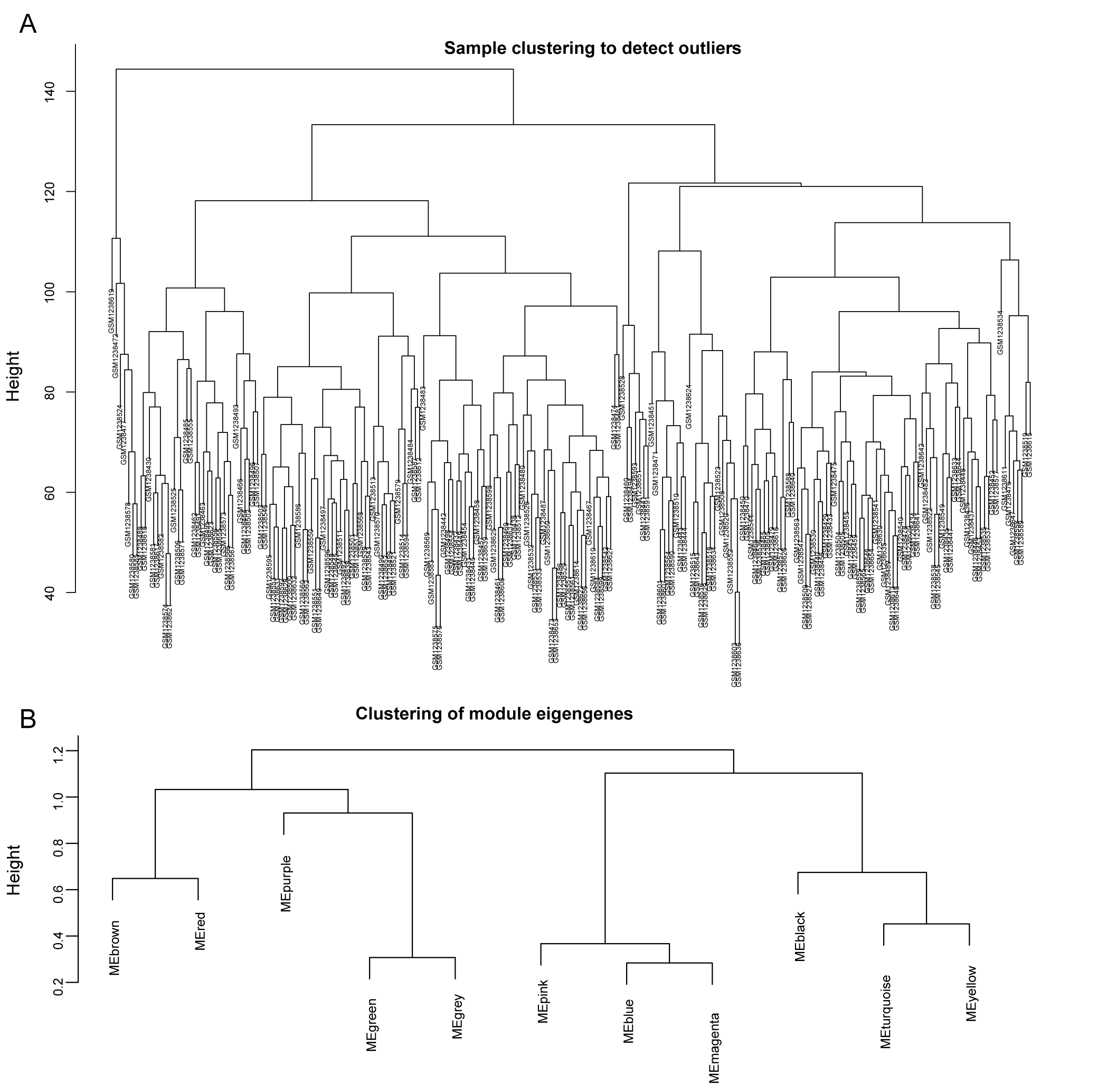

Supplement: Supplementary file 2 — Supplementary file2 (TIF 14098 KB) [file 10067_2024_6901_MOESM2_ESM.tif]

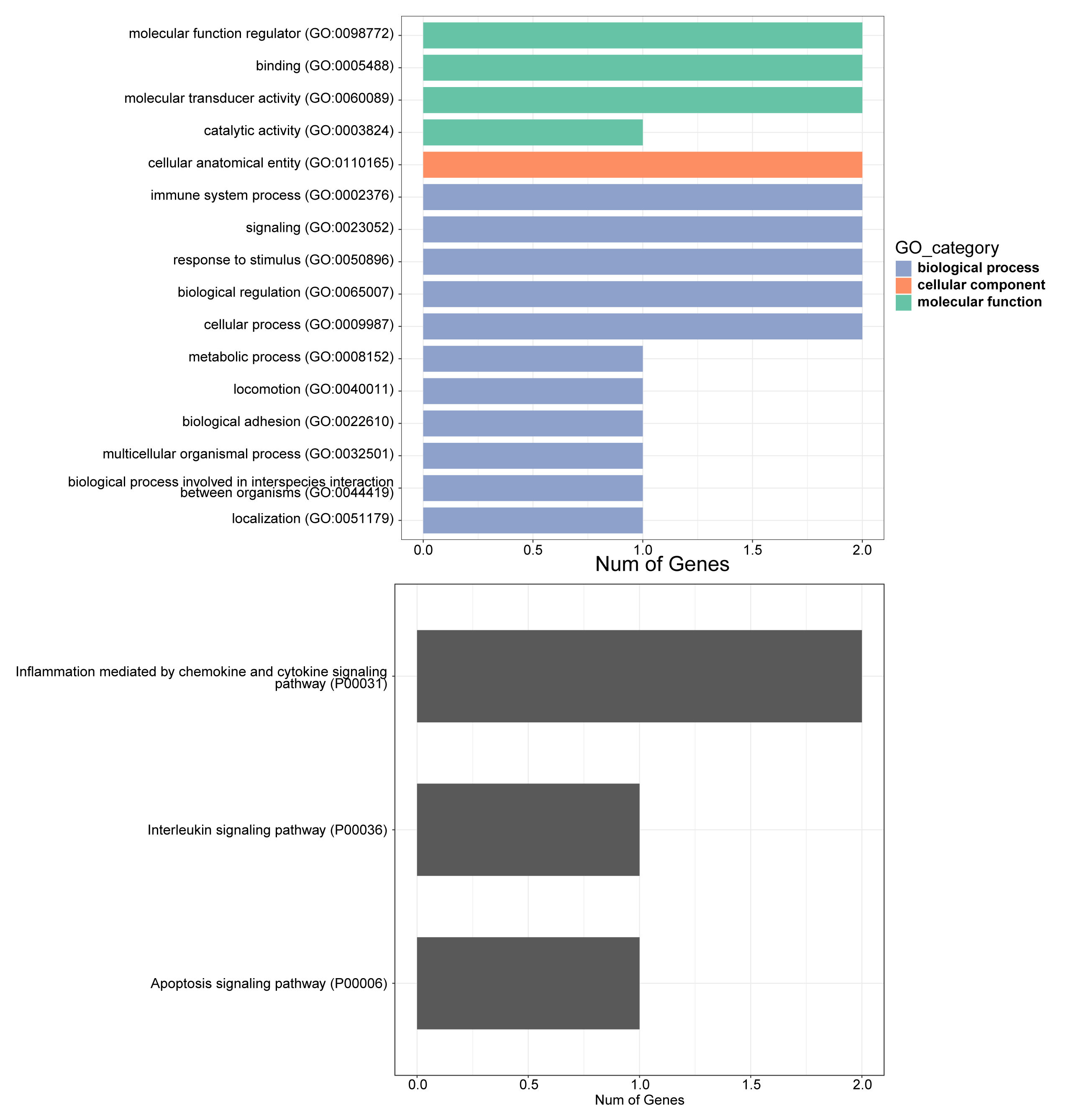

Supplement: Supplementary file 3 — Supplementary file3 (TIF 21525 KB) [file 10067_2024_6901_MOESM3_ESM.tif]

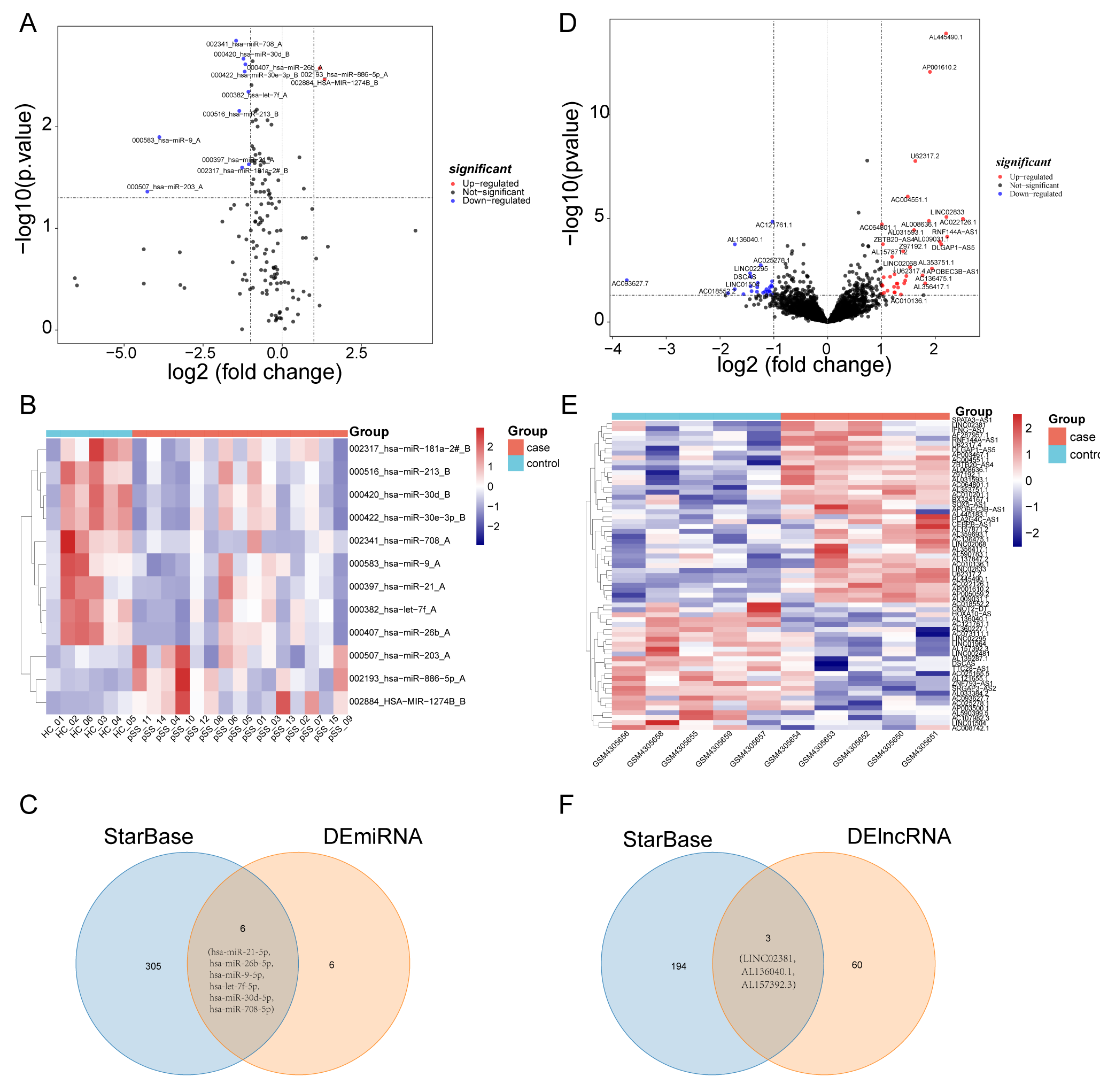

Supplement: Supplementary file 4 — Supplementary file4 (TIF 13542 KB) [file 10067_2024_6901_MOESM4_ESM.tif]
